# Box Office Forecasting considering Competitive Environment and Word-of-Mouth in Social Networks: A Case Study of Korean Film Market

**DOI:** 10.1155/2017/4315419

**Published:** 2017-07-27

**Authors:** Taegu Kim, Jungsik Hong, Pilsung Kang

**Affiliations:** ^1^Department of Industrial and Management Engineering, Hanbat National University, Daejeon, Republic of Korea; ^2^Department of Industrial and Systems Engineering, Seoul National University of Science and Technology, Seoul, Republic of Korea; ^3^School of Industrial Management Engineering, Korea University, Seoul, Republic of Korea

## Abstract

Accurate box office forecasting models are developed by considering competition and word-of-mouth (WOM) effects in addition to screening-related information. Nationality, genre, ratings, and distributors of motion pictures running concurrently with the target motion picture are used to describe the competition, whereas the numbers of informative, positive, and negative mentions posted on social network services (SNS) are used to gauge the atmosphere spread by WOM. Among these candidate variables, only significant variables are selected by genetic algorithm (GA), based on which machine learning algorithms are trained to build forecasting models. The forecasts are combined to improve forecasting performance. Experimental results on the Korean film market show that the forecasting accuracy in early screening periods can be significantly improved by considering competition. In addition, WOM has a stronger influence on total box office forecasting. Considering both competition and WOM improves forecasting performance to a larger extent than when only one of them is considered.

## 1. Introduction

The magic of movies and their hold on the masses have made the motion picture industry one of the main sectors of today's global economy [1, 2]. With numerous new movie releases around the globe every year, global box office earnings increased to $34.7 billion in 2012, up to 6% over the previous year [3]. Moreover, the film market is expected to experience constant growth up to 2017 [4]. Despite their inherent charms and promising prospects at the industry level, investing in motion pictures is still considered to be a high risk business for film makers, distributors, and even exhibitors, because of their alarming failure rates at the individual motion picture level [5, 6].

This high risk-high returns situation has consequently led many researchers to attempt the interesting and challenging task of forecasting the box office [7]. Accurate forecasts benefit the entire value chain in many ways, from enabling more informed investment decisions to planning better marketing and entry time strategies. However, having accurate forecasts in the early stages of a movie is very difficult owing to a distinctive characteristic of motion pictures. Because a movie is an experiential product, a consumer has limited access to information about it before he or she actually watches it. Hence, the degree of satisfaction derived from a particular movie, that is, a possible proxy of future prospects, can be evaluated only after watching it. Consequently, an accurate prediction for its final success or failure can hardly be made until the audience has accumulated to a certain level [8, 9].

To resolve the practical importance and inherent difficulty described above, a number of studies have been devoted to forecasting the box office. They can be divided into three categories on the basis of their main research subjects: (1) identifying and investigating influential factors or explanatory variables in forecasting models, (2) introducing new forecasting algorithms, and (3) analyzing the effects of different forecasting horizons. Regarding the first subject category, a number of studies have attempted to determine significant factors and assess their impacts on or their relative importance in box office forecasting. Among the numerous explanatory variables tested, the following three types of factors have been found to be significant: (i) factors concerning movie characteristics, that is, screening statistics data, such as the star, director, genre, sequels, ratings, distributors, production, and marketing budgets, and the number of screens (or screening schedules) [10–16], (ii) competition factors reflecting market conditions, such as the number of existing and newly introduced movies [11, 17, 18], and (iii) word-of-mouth (WOM) effects, such as the intensity of interest and the level of preference derived from user ratings and social network services (SNS) [18–22].

Regarding the second subject category, most proposed forecasting algorithms fall into one of the following three subgroups: (a) a statistical learning model, such as linear regression and probabilistic models [1, 10, 13, 23–26], (b) time-series forecasting models, such as diffusion models and the vector autoregression method [27–30], and (c) sophisticated machine learning-based models, such as artificial neural networks (ANN) [12, 31].

Regarding the last subject category, analysts tend to use the time near the release date as the forecast horizon. In particular, early forecasting is the most valuable leverage to timely and effective managerial decisions, because the box office score from the opening weekend and the days that follow forms a significant portion of the movie's overall success [23]. Consequently, many studies have focused on developing “before release” or “at release” forecasting models [13, 32].

Although the aforementioned subjects have been individually investigated by a large number of studies, little has been done to systematically address all these topics simultaneously from a modeling perspective. Therefore, in this paper, we attempt to build accurate box office forecasting model by incorporating various factors in the model's definition and by adopting well-known machine learning-based nonlinear regression algorithms in model building. Additionally, we attempt to systematically investigate the contributions of different factor categories to forecasting accuracy improvements for different forecasting horizons by decomposing the performance improvements into four sequences under two possible scenarios.  Table 1 summarizes the main contributions of this paper in terms of model design, building, and interpretation along with the three mainstreams in box office forecasting research.

In order to improve the forecasting accuracy, a large number of explanatory variables from three different categories are identified. The first variable category concerns screening information. As a screening-related variable, the number of seats scheduled at the release date, which is the most commonly used proxy of the distribution power, is selected. The second variable category is the competition environment. Movies with overlapped screening periods are in a zero-sum competition for limited audiences. Therefore, the release strategy (comprising the managerial decisions to comprehend the competitive condition of the current market and to determine when the movie should be released) has significantly influenced the final box office score. Although many researchers have shed light on this issue and have referred to it as an “entry strategy” [33] or a “timing game” [34], there is little agreement on how the overall competitive environment should be quantified. In this study, we create as many variables as possible, ensuring that they are publicly available from reliable sources on the web. In doing so, we expect to eliminate unintentional human biases that are caused by arbitrary definitions or the varying methods of measuring competition variables used in previous studies. The third variable category is the effect of WOM. As noted before, a number of attempts exploiting WOM effects in box office forecasting have been made, because a film is defined as an experiential and cultural product shared by social human beings. Contrary to the previous studies in which WOM-related variables were obtained from interviews, random dialing surveys, online user ratings, or even simulation experiments [1, 28, 35, 36], recent studies have tended to use SNS as a more transparent, authentic, and consumer-driven source of WOM [19, 37] in order to overcome the limitations of the aforementioned methods, such as high costs and sampling biases. As an extension of our previous study [38], we utilize WOM-related variables by collecting various types of mentions referring to the target motion picture posted on SNS. We should note that the abundant data available online allow us to expand the range of the aforementioned candidate explanatory variables without much cost. Competition-related variables can be obtained from an authorized association of the Korean motion picture industry, whereas WOM-related variables can be obtained from various SNS websites or applications. Once all the candidate explanatory variables are identified and relevant measures collected, a genetic algorithm (GA) is used to identify significant variables in the model building step. By employing the GA, an explanatory variable's significance is determined during the algorithm training process so as to eliminate human selection biases.

In order to improve forecasting accuracy during model building, we adopt machine learning-based nonlinear algorithms that can capture both linear and nonlinear relationships between the explanatory variables and box office scores. Since a large number of candidate explanatory variables and different forecasting time horizons are considered, the assumption of a multivariate linear regression—the relationship between the explanatory variables and the target variable is always linear—may often be violated in practice. In this situation, it is more suitable to increase the model's complexity by employing more sophisticated regression algorithms. Another remedy to improve forecasting accuracy includes constructing forecasts combinations by aggregating forecasts made by machine learning-based models.

The third main contribution of this paper is to exploit the individual influence of each remedy, that is, considering diverse factors, employing machine learning algorithms, and constructing forecasts combinations, under different forecasting horizons. In order to do so, we set three different forecasting horizons: (1) one week after release, (2) two weeks after release, and (3) total box office. For each forecasting horizon, two scenarios are generated under which forecasting accuracy improvements are decomposed into four sequences. In the first scenario, the effect of competition is analyzed followed by analyses of the effects of WOM, machine learning algorithms, and forecasting combinations. In the second scenario, the first two sequences are reversed; the effect of WOM is first analyzed, followed by the effects of competition, machine learning algorithms, and forecasting combinations.

In summary, the ultimate goal of this paper is to develop a more accurate box office forecasting model at the release date by using machine learning-based algorithms and considering competitive environments and WOM effects simultaneously. We also employ a combination of individual forecasting models to enhance forecasting accuracy. Incorporating a wide range of variables would help not only to improve model performance but also to comprehend the characteristics of specific factors, particularly the dynamics of competition over time. The proposed model is expected to contribute to efficient and timely managerial decisions, such as initial screen scheduling for exhibitors, entry time strategy for distributors, and marketing budget allocation.

The rest of this paper is organized as follows.  Section 2 demonstrates the proposed box office forecasting model including data description and collection, variable selection, forecasting models, and performance criteria.  Section 3 validates the proposed model in terms of forecasting accuracy and analyzes the effects of different factors in different time horizons.  Section 4 concludes and outlines future research directions.

## 2. Methodology

**Figure 1:**
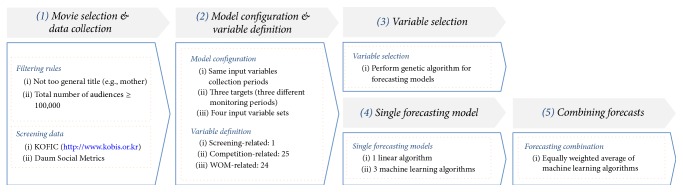
Research framework for developing box office forecasting models.

The overall research framework is illustrated in Figure 1. In the first step, motion pictures for building forecasting models are selected using three filtering rules: (1) time horizon for the analysis, (2) existence of general words in the title, and (3) the minimum total audience. For the selected motion pictures, a total of 50 variables from 3 categories are collected: one from screening data, 25 from competition-related data, and 24 from WOM-related data. In the second step, the structures of the three forecasting models are defined: when and what to forecast and which variables to use. In this study, all three forecasting models use the explanatory variables collected from an identical period; the difference among the models is the monitoring period for the target variable. Model W_1_ and Model W_2_ predict the cumulative box office takings in the first and second week after the release date, respectively, whereas Model T predicts the total cumulative box office takings over the entire screening period. In the third and fourth steps, significant explanatory variables are selected using the GA, and only these selected variables are used for training forecasting algorithms. As forecasting algorithms, one linear model (the MLR) and three machine learning-based NLR algorithms (support vector regression (SVR), Gaussian process regression (GPR), and* k*-nearest neighbor regression (*k*-NN)) are employed. In the final step, a combination of forecasts made by individual nonlinear algorithms is constructed to improve the forecasting accuracy.

### 2.1. Motion Picture Selection and Candidate Explanatory Variable Identification

Owing to the data availability issue, we first collect screening-related information for motion pictures released in the recent two and half years. Among them, the motion pictures that do not satisfy the following two criteria are removed from consideration: (1) the total audience exceeds 100,000, and (2) the title is not a single general word, such as “war” or “mother.” The first filtering rule is set because it is practically impossible to gather significant amount of SNS data for unpopular motion pictures, which would result in unfair comparisons among the motion pictures being considered in terms of WOM effects. On the other hand, the second filtering rule is used to avoid excessive collection of irrelevant SNS data. For example, one of the more successful motion pictures in our data collection period includes the word “mother” in the Korean letter. It is not difficult to collect SNS mentions or blog posts discussing this term, but identifying whether those mentions or posts actually refer to the motion picture titled “mother” is a cumbersome text mining-related issue. If this issue cannot be resolved satisfactorily, the forecasting model would not perform as desired. Thus, it is better not to consider such motion pictures until the precision of text mining technologies reaches a certain level. After applying the abovementioned filtering rules, a total of 175 motion pictures are finally selected for further analysis.

As an attempt to construct a complete and reliable set of candidate explanatory variables, we collect data that meet the following three criteria: (1) identify as many variables as possible, (2) ensure that the identified variables are measurable and collectable from reliable sources, and (3) ensure that the data collection processes are objective and unbiased. As a result, a total of 50 explanatory variables from 3 different categories are identified and collected as shown in Table 2. Screening-related variables, which include the number of seats and screens and the screen share on the release day, are collected from the Korean Film Council [39]. It should be noted that these three variables contain almost identical information, and thus, they are highly correlated. Moreover, the level of abstraction increases in the order of the number of seats, the number of screens, and the screen share. Thus, we decide to use the most detailed information, that is, the total number of seats (Var2), as the representative screening-related variable.

A total of 25 variables collected from the same source as the screening variables comprise the competition-related variables. In order to consider the competition environment, the top five motion pictures in terms of box office takings one day prior to the release of the target motion picture are first selected. Let us denote the target motion picture as* X*, whereas *Top*_1_ and *Top*_1–5_ refer to the top-ranked motion picture and the top five motion pictures one day before the release date, respectively. Previous studies have verified that the box office scores of a newly released motion picture are affected by its competition structure with other motion pictures exhibited in the concurrent period, in terms of how many of these other pictures have the same genre, ratings, nationality, and distributors as the target motion picture [11, 18, 31, 40, 41]. Therefore, the numbers of seats and screens and the screen share of *Top*_1_ on the release day as well as one day before the release date are collected to investigate the effect of the current blockbuster on newly released motion pictures (Var3~Var8). In addition, the difference of the number of seats for *Top*_1_ between the release day and one day before release is derived to consider the indirect effect of the distribution power of* X* on *Top*_1_ (Var27). In order to investigate the overall competition environment, the number of motion pictures, total screens, total seats, and aggregated screen shares of *Top*_1–5_ with the same nationality, genre, ratings, and distributors as* X* are collected (Var9~Var24). In addition, the average screening days of *T*_1–5_ until release (Var25) are also considered to exploit the opening effect of the competitors, whereas the rank of* X* in terms of the number of screens (Var26) is considered to gauge the initial marketing power of* X*.

As WOM-related variables, a total of 24 variables are collected from Daum Social Metrics (http://www.socialmetrics.co.kr), a social information service company that collects SNS data from various SNS sites, such as Twitter and Facebook, and provides summarized SNS data for a given query after conducting a sentiment and polarity analysis. The time horizon of SNS data collection is three weeks in our study; that is, we initially consider SNS mentions referring to the target motion picture that are created from three weeks prior to its release to one day before its release. Then, on the basis of the characteristics of the mentions or posts (e.g., whether it is informative, or whether its nuance is positive), the numbers of total, emotional, positive, and negative mentions in each week are recorded (Var28~Var39). Then, using these original SNS data, the total numbers, average increases, and weekly increases in the total, emotional, positive, and negative mentions are derived, respectively (Var40~Var51).

### 2.2. Model Configuration

Since the main purpose of this study is to improve forecasting performance by considering competition structure and WOM effects and by adopting machine learning-based nonlinear algorithms, forecasting models can be distinguished using three criteria: target data collection period, explanatory variable construction, and forecasting algorithms. Based on these criteria, the following notation is used in the remainder of this paper.

  Model* [Target Period] (Explanatory Variables, Forecasting Algorithm)*

The descriptions of the identifiers for each criterion are provided in Table 3. Because screens and seats are perishable, it is very important to have accurate forecasting in the early stage of the screening period, for exhibitors to allocate the appropriate number of screens to each running motion picture, and thus to maximize their profits [13, 23, 32]. Therefore, we determine that three targets need to be forecasted: the accumulated box office takings in the first week and in the first two weeks and for the entire screening period. Although it is possible to set a daily box office score as a target variable, the seasonality issue must be resolved during the modeling. Hence, weekly box offices have been commonly used target variables in many previous studies [18, 41].

In terms of explanatory variables, we construct four different explanatory variable sets to analyze the direct effect of each category: screening variable only (S), screening with competition variables (SC), screening with WOM variables (SW), and screening, competition, and WOM variables together (SCW). As forecasting algorithms, four regression algorithms, that is, MLR, SVR, GPR, and* k*-NN, and the combination of the latter three algorithms are used. A brief description of each of these forecasting algorithms appears in Section 2.4. Note that all four explanatory variable sets are used for MLR, but only the last set, that is, wherein all three categories are combined, is used for the machine learning-based algorithms (SVR, GPR, and* k*-NN). The tested combinations of the explanatory variable set-forecasting algorithm are provided in Table 4.

**Figure 2:**
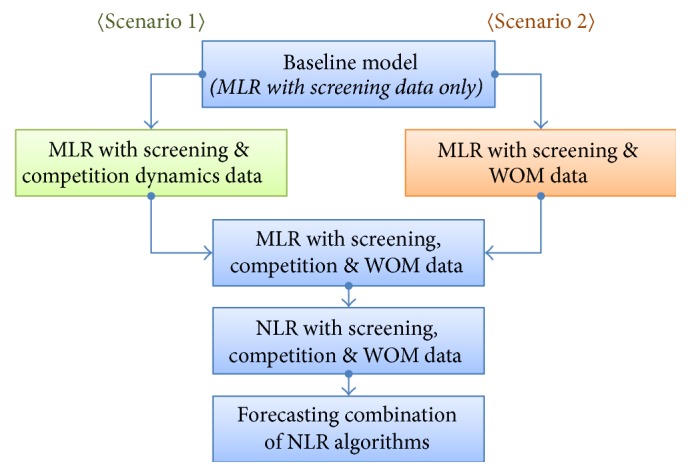
Two scenarios for analyzing forecasting performance improvement.

In order to verify the effect of each variable category, we build two hypothetical scenarios as shown in Figure 2, under which forecasting performance improvements can be decomposed based on the usage of variable categories and type of forecasting algorithm. Under scenario 1, we trace performance improvements in the following sequence: considering the competition environment, considering both competition environment and WOM effects, adopting machine learning-based NLR forecasting algorithms with all possible variable categories, and considering the combination of NLR algorithms. Scenario 2 differs in one sense only: the first two steps are reversed; the WOM effect is investigated first, followed by the examination of both competition environment and WOM effects.

### 2.3. Variable Selection

A maximum of 50 candidate explanatory variables from 3 categories are allowable while building forecasting models, which is relatively high compared to the total number of motion pictures. Since too many explanatory variables may sometimes degenerate forecasting accuracy, it is necessary to select a set of significant variables to ensure that the forecasting model is both effective and efficient. Thus, we adopted the GA for the variable selection method. GA is typically used to find a pseudo-optimal set of explanatory variables by reproducing natural evolutionary procedures such as selection, crossover, and mutation [42–44]. In a GA variable selection, a sufficient number of chromosomes, called a population, are initially generated. Each chromosome is represented as a vector having the same length of the total number of variables. Each cell in a chromosome, called a gene, has a value of either 1 or 0, which designates whether the corresponding variable is activated during modeling (1) or not (0). Forecasting algorithms are trained based on activated variables determined by each chromosome, and their fitness values, that is, mean absolute percentage error (MAPE), are calculated. Then, the chromosomes with higher fitness values survive (selection) to produce the next generation of chromosomes by exchanging some part of their genes (crossover) and reversing the gene value with a very low probability (mutation) so as to have an opportunity to escape from the local optimum. Hence, by iterating the selection-crossover-mutation cycle a sufficient number of times, a pseudo-optimal set of explanatory variables can be identified. We used probabilistic crossover with the crossover rate of 0.5 and the mutation rate was set to 0.05. In addition, the population size was set to 50.

### 2.4. Forecasting Models

As explained in Section 3.2, four regression algorithms are adopted to build box office forecasting models: MLR, SVR, GPR, and* k*-NN. The MLR [45] fits the linear functional relationship between the multiple explanatory variables and the target variable of the given training data. Due to its theoretically sound foundations, it has been most widely adopted as a forecasting algorithm in many applications [46–49]. The SVR [50] is a regression version of the support vector machine (SVM) that is based on the structural risk minimization (SRM) principle. The SVR finds the regression equation $\hat{y}=w^{T}x+b$ that meets the following two goals: (1) the regression function should be as flat as possible to achieve generality, and (2) to the extent possible, training instances should be within the *ε*-tube so as to minimize the prediction error. The SVR can result in higher forecasting accuracy by allowing nonlinear fitting using a mapping function that transforms the original explanatory data in a low-dimensional space into a high-dimensional feature space. The GPR [51] begins with the MLR equation and applies the Bayesian approach and kernel tricks in order to extend its expressiveness. In the GPR, the target variable *y* is expressed as a linear combination of the explanatory variables with a Gaussian noise. Inference in the GPR is conducted based on the posterior distribution over the weights by applying Bayes' theorem. As in the SVR, the GPR can find a nonlinear relationship by introducing a kernel mapping function that projects the explanatory data from a low-dimensional space to a higher dimensional feature space and by using kernel tricks to compute inner products in the feature space. The* k*-NN [52], also called case-based or memory-based reasoning, is the most popular instance-based learning algorithm. Since it does not require a separate training procedure, it has been successfully employed in various domains where rapid and frequent model updates are required [53, 54]. The* k*-NN makes a forecast for a new motion picture on the basis of similarities among the explanatory variables with its close neighbors. Once a test motion picture *x*_*t*_ is provided,* k*-NN first searches the *k* most similar motion pictures in the reference data set using a certain similarity metric for a set of explanatory variables. Then, the box offices of neighbor motion pictures are aggregated based on a certain weight allocation method.

In addition to individual regression algorithms, the combination of three machine learning-based regression algorithms is constructed to improve the forecasting accuracy. Firstly, each machine learning-based algorithm, that is, SVR, GPR, and* k*-NN, is trained with its best parameters and selected significant variables. Then, for each motion picture, three forecasts made by the three algorithms are combined to produce a single output using the equally weighted average method.

### 2.5. Validation Method and Performance Measure

Since the SVR, GPR, and* k*-NN need optimization of algorithm-specific parameters (type of kernel, kernel specification, and error cost for the SVR; kernel width and hyperparameters for prior distribution for the GPR; and number of nearest neighbors for the* k*-NN), 10-fold cross-validation is conducted to select the best parameter combinations for each forecasting algorithm [55]. In a 10-fold cross-validation, the entire data set is divided into 10 segments. Each segment is reserved in turn for validation, and the rest are used for training the algorithm. The validation results from 10 segments under an identical parameter setting are then aggregated to evaluate performance with the corresponding parameter values. The most accurate parameter setting is used for further analysis. Once the parameters are settled, the performances of the forecasting algorithms are evaluated and compared using the leave-one-out method [56]. It is the most fragmented form of* k*-fold cross-validation, because *k* is set to the total number of motion pictures. When the number of training samples is insufficient, the leave-one-out test method can produce better forecasting performance, provided as many training samples as possible are secured.

Once the forecasting algorithms are trained and forecasts for all motion pictures are made, each forecasting algorithm is evaluated in terms of MAPE as follows:

(1) $$\begin{array}{c} \text{MAPE}=\frac{1}{n}\overset{n}{\underset{i=1}{\sum }}\left|\;\frac{y_{i}-{\hat{y}}_{i}}{y_{i}}\;\right|, \end{array}$$

where *y*_*i*_ and ${\hat{y}}_{i}$ are the actual box office score and the forecast of *i*th motion picture made by the model, respectively.

## 3. Results

### 3.1. Variable Selection

The selected explanatory variables for three different targets using the MLR as a base learner are summarized in Table 5. For all targets, the only screening variable, that is, the number of seats for* X* (*N*_seat^*x*^_*r*_), is found to be significant in forecasting the box office. Among competition-related variables, it is found that either the number of screens or the number of seats for *Top*_1_ is a critical factor for an accurate box office forecast for* X* across all periods. When looking at each model individually, the numbers, screens, and seats for the motion pictures in *Top*_1–5_ with the same nationality, genre, ratings, and distributors have significant influences on the box office forecast for Model W_1_(SC, MLR). An interesting observation is that neither the average age of competitors nor the rank of* X* in terms of the number of screens on the release day has a significant influence on the box office in that period. Regarding Model W_1_(SW, MLR), five WOM-related variables are determined as being significant:* N*_emo_−1_,* N*_pos_−1_, Weekly_SNS_inc, Tot_emo, and Weekly_pos_inc. It is worth noting that, among the five WOM-related variables, four are related to the volume of emotional mentions, especially positive ones; it can be expected that, in the early stage of screening, the impact of a positive WOM atmosphere overwhelms negative WOM effects.

For the cumulative box office forecast during the first two weeks using competition-related variables (Model W_2_(SC, MLR)), we see results identical to those of Model W_1_(SC, MLR) in every way except one. Two additional competition-related variables are included: Sc_share^*y*^_*r*_ and Avg_age. Although the average age of competitors is not determined as being significant for Model W_1_(SC, MLR), it becomes a critical factor when the monitoring period increases. Since a common box office distribution over the entire screening period is positively skewed, that is, most of the total revenues are accumulated in early screening periods, the ticketing power of competitors may shrink before the target motion picture begins to lose ticketing power due to the time lags between the release dates. Therefore, it could be interpreted that, during the second week,* X* still has a considerable opening effect, but it diminishes for those in *Top*_1–5_. For Model W_2_(SW, MLR), six WOM-related variables are identified as being significant. Interestingly, (1) none of these variables are selected for Model W_1_(SW, MLR), and (2) two negative WOM variables are included.

Regarding Model T(SC, MLR), that is, forecasting the total cumulative box office using competition-related variables, most of the selected variables are identical to those selected for either Model W_1_(SC, MLR) or Model W_2_(SC, MLR). A noticeable difference is the inclusion of the number of seats and its changes for *Top*_1_ (*N*_seat^*y*^_*r*_, *N*_seat^*y*^_*r*−1_, and *N*seat_increase^*y*^) and the rank of* X* (Rank_screen) in terms of the number of screens on the release day. It can be interpreted that (1) if a successful blockbuster is running when* X* is released, it may affect the box office of* X* over the entire period and (2) the marketing power of* X* at the beginning of screening is highly proportional to the success of the total box office, although it may not be significantly relevant to the early screening periods. For Model T(SW, MLR), more variables are selected compared to Model W_1_(SW, MLR) and Model W_2_(SW, MLR); a total of 10 variables are found to be significant. It should also be noted that the informative mentions do not affect the forecast of the total cumulative box office; all the selected variables are emotional: positive, negative, or the sum of both.

Table 6 summarizes the number of selected variables in each category for each forecasting model and algorithm when all the screening, competition, and WOM-related variables are considered together, whereas the detailed selection results are provided in Table 7. Since the total number of candidate variables is 50, it would be time consuming to discuss whether each variable is selected for each forecasting model. Instead, it would be more worthwhile to derive common trends in variable selection results. Firstly, the only screening-related variable is always selected irrespective of the target definitions and forecasting algorithms. It implies that although additional information is available, the screening information is the fundamental source of box office forecasting and cannot be replaced by any type of information. Secondly, in general, approximately half the candidate variables are identified as being significant. The smallest and largest number of explanatory variables is 18 for Model W_1_(SCW, SVR) and 26 for Model W_2_(SCW,* k*-NN), respectively. Lastly, when looking at the variable sources separately, competition-related variables are favored selections for machine learning-based regression algorithms in early monitoring periods (Model W_1_ and Model W_2_), whereas WOM-related variables are favored selections over the entire period (Model T).

### 3.2. Forecasting Accuracy

**Figure 3:**
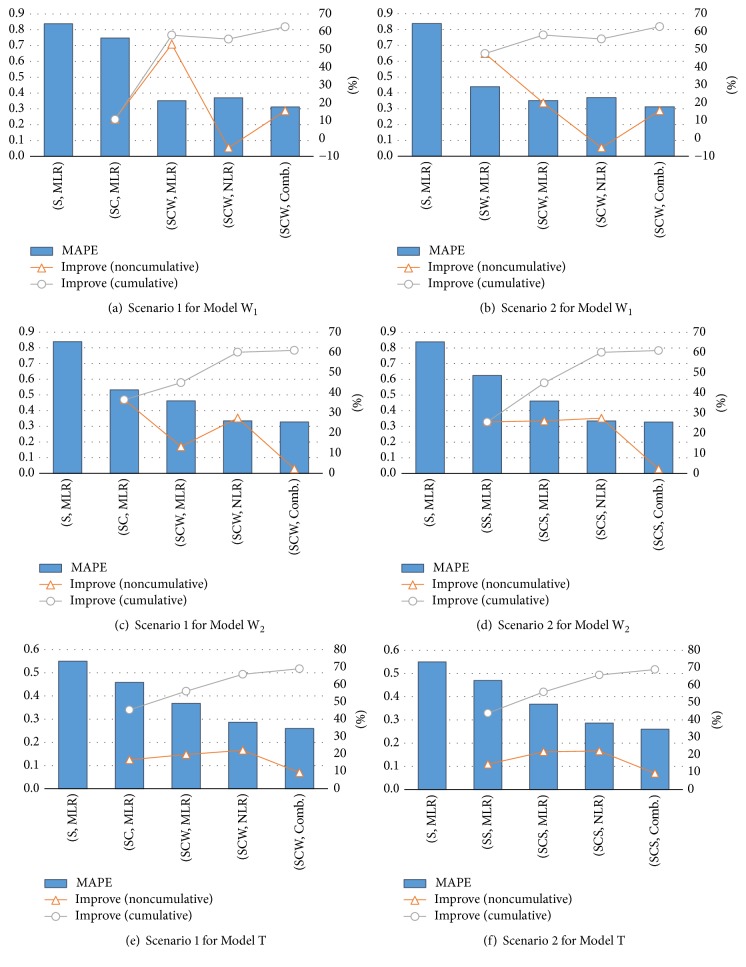
Performance improvements in two different scenarios for three forecasting models (MAPE: bar chart, *y*-axis on the left, improve: line graph, *y*-axis on the right).

Table 8 summarizes the forecasting performances of all models in terms of the MAPE and the improvements and their statistical significance against (S, MLR), (SC, MLR), (SW, MLR), and (SCW, MLR). In addition, the performance improvements achieved by considering the additional category of explanatory variables under two different scenarios are also illustrated in Figure 3. Under scenario 1, the effect of competition-related variables on forecast accuracy is first investigated, and the analysis of the WOM-related variables follows. Under scenario 2, on the other hand, the effect of WOM-related variables on forecast accuracy is investigated first, followed by the analysis of the competition-related variables. The rest of the analysis steps are identical for both scenarios; the collaborative effect of the competition environment and WOM is exploited with the MLR, followed by verification of the effects of machine learning-based regression algorithms and their combinations on forecasting accuracy improvements.

For Model W_1_, the MAPE of the forecasts made by the MLR with the screening variable only (Model W_1_(S, MLR)) is 0.8383. In other words, the relative difference between the forecast and the actual cumulative box office in the first running week is greater than 83% on average, which, in practice, is not conductive for supporting managerial decisions. However, the MAPE of Model W_1_ is reduced when either competition-related variables (Model W_1_(SC, MLR)) or WOM-related variables (Model W_1_(SW, MLR)) are taken into consideration. Further, the effect of competition-related variables is more dramatic than that of WOM-related variables; the MAPE of Model W_1_(SC, MLR) improves by 47.64% over that of Model W_1_(S, MLR), and the improvement is statistically significant at *α* = 0.05, whereas the MAPE of Model W_1_(SW, MLR) improves by 10.75%. Based on this result, it can be concluded that, in the early periods of screening, the competition environment around the target motion picture, that is, the characteristics of other motion pictures running concurrently with the target motion picture, dominantly affects its success, whereas the WOM that is spread prior to release has little influence on its box office performance. Once both the competition and the WOM variables are taken into account, however, the forecasting accuracy can be improved even further; the MAPE of Model W_1_(SCW, MLR) is 0.3515 and is improved by 19.92% and 53.02% over that of Model W_1_(SC, MLR) and Model W_1_(SW, MLR), respectively. In other words, a significant synergy effect may be achieved when competition-related variables and WOM-related variables are utilized together in forecasting the box office. When looking at the forecasting accuracies of machine learning-based algorithms, however, the improvement is not significant for Model W_1_; two algorithms (GPR and* k*-NN) result in lower MAPEs than the MLR, but their improvements are marginal. Further, the SVR even degrades the forecasting accuracy by 25%, which gives rise to a higher average MAPE for machine learning-based algorithms. Although individual machine learning-based algorithms do not succeed in significant forecasting performance improvements, a combination of these algorithms provides assured improvements; the MAPE of Model W_1_(SCW, Comb.) is 0.3121 and is statistically lower than that of Model W_1_(S, MLR), Model W_1_(SC, MLR), and Model W_1_(SW, MLR). Although the improvement in the MAPE of Model W_1_(SCW, Comb.) against that of Model W_1_(SCW, MLR) is not statistically significant, it exceeds 10% (11.20%), which is quite high.

Regarding Model W_2_, the MAPE of Model W_2_(S, MLR) is 0.8391 and is as inaccurate as that of Model W_1_(S, MLR). An interesting observation is that, unlike Model W_1_, the contribution of WOM to forecasting accuracy improvement is greater than that of the competition environment; the MAPEs of Model W_2_(SW, MLR) and Model W_2_(SC, MLR) are 0.5325 and 0.6245, respectively, and the corresponding improvements against Model W_2_(S, MLR) are 36.54% and 25.58%, respectively. These forecasting performance improvements imply that the competition environment is the primary factor affecting the success of a newly released motion picture for a relatively short period from the release date, whereas the effect of WOM becomes dominant when the monitoring period increases. Similar to Model W_1_, forecasting performance can be further improved when the competition environment and WOM are considered simultaneously, compared to incorporating each category separately; the MAPE of Model W_2_(SCW, MLR) is 0.4616 and is improved by 26.08% and 13.30% against that of Model W_2_(SC, MLR) and Model W_2_(SW, MLR), respectively. It is worth noting that, unlike Model W_1_, forecasting performance improvements made by machine learning-based algorithms are impressive; all the three algorithms (Model W_2_(SCW, SVR), Model W_2_(SCW, GPR), and Model W_2_(SCW,* k*-NN)) improved the MAPE by more than 55% over that of Model W_2_(S, MLR), by more than 40% against that of Model W_2_(SC, MLR), and by more than 29% against Model W_2_(SCW, MLR). Moreover, all the improvements are statistically significant at *α* = 0.2. Since the variation of the target values, that is, the cumulative box office, becomes larger as the monitoring period increases, the relationship between the explanatory variables and the target variable cannot be fully captured by a simple linear model. However, some portions of those missed relationships can be explained by nonlinear algorithms, such as the SVR, GPR, and* k*-NN, and they may lower MAPE values. In addition, the effect of forecasts combination is also significant for Model W_2_; the MAPE of Model W_2_(SCW, Comb.) is improved by 29.08% against that of Model W_2_(SCW, MLR).

For Model T, a relatively lower MAPE can be achieved by the baseline model compared to the other two targets; the MAPE of Model T(S, MLR) is 0.5501, whereas those of W_1_(S, MLR) and W_2_(S, MLR) are 0.8383 and 0.8391, respectively. This result can be attributed to the way the MAPE is computed; it does not measure the extent of the difference between the actual and predicted targets. Rather, it measures the relative difference in terms of the actual target value. Therefore, although the difference between the actual and predicted target values increases, it is possible to obtain a lower MAPE if the actual target value increases significantly. For example, assume that the actual and predicted box office takings are 10 million and 11 million units, respectively. Then, the MAPE becomes 0.1. Later, if the total actual and predicted box office takings become 100 million and 105 million, respectively, then the MAPE decreases to 0.05, although the absolute difference increases from 1 million to 5 million. Indeed, this helps us explain the MAPE of Model T; the increased denominator in (1) results in a lower MAPE. Similarly, Model T(SC, MLR) and Model T(SW, MLR) provide lower MAPEs with identical explanatory variables and forecasting algorithms but different targets. Regarding the effect of competition and WOM, they almost equally contribute to forecasting performance improvements; the MAPE of Model T(SC, MLR) is 0.4702 and is improved by 14.54% against that of Model T(S, MLR), whereas the MAPE of Model T(SW, MLR) is 0.4583 and is improved by 16.69% against that of Model T(S, MLR). When competition and WOM variables are simultaneously considered, additional improvements can be achieved; the MAPE of Model T(SCW, MLR) is 0.3681 and is decreased by approximately 20% against that of the model with only one of the two variable categories. In addition, utilizing machine learning-based nonlinear forecasting algorithms even lowered the MAPE by at least 15% compared to that of Model T(SCW, MLR). Finally, the MAPE can be improved by an additional 10% by combining nonlinear algorithms; the MAPE of Model T(SCW, Comb.) is 0.2599, the lowest among all forecasting models.

In summary, the following observations can be derived from the experimental results. Firstly, incorporating the competition environment or WOM effects into modeling certainly improves forecasting accuracy in terms of the MAPE for all monitoring periods. In addition, these two categories demonstrate a significant synergy when they are considered simultaneously. The competition environment exerts a greater influence in the early stage of screening, whereas WOM effects have a greater influence for a longer period. Second, there are some differences among the significant variables in each category according to the monitoring period; the average age is found to be critical after at least one week of the running period, whereas the proximity of the distribution power of the target motion picture, that is, the rank of the number of screens on the release day, is found to be critical for the total box office, but not for the box office in the early periods. Among the WOM variables, while none of the negative mentions are critical in the earlier periods, they become significant when the monitoring period increased. In addition, as the monitoring period becomes wider, more WOM variables are required for forecasting models. Regarding the forecasting algorithms, machine learning-based nonlinear models generally improve the linear model (MLR), with only one exception. Further, the combination of these nonlinear algorithms even enhances forecasting performance; the MAPEs of the forecasts combinations are at least 10% lower than those of the MLR with the same variable utilization for all monitoring periods.

## 4. Conclusion

In this paper, we attempted to improve the accuracy of box office forecasts by considering a wide range of competition and WOM-related factors with the help of well-known machine learning-based NLR algorithms. Furthermore, we also systematically investigated the contributions of different factor categories to forecasting accuracy improvements in different forecasting horizons by decomposing the performance improvements into four sequences under two possible scenarios. The experimental results showed that considering both the competition environment and WOM effects improved forecasting accuracy. Although their degrees of contribution differed according to the monitoring periods, we could always verify a significant synergy for all periods. Notably, the box office score in the early stages was highly affected by the competition environment, whereas the influence of WOM was greater than the competition environment when forecasting the total box office score. With regard to forecasting algorithms, machine learning-based NLR algorithms can achieve higher forecasting accuracies than traditional MLRs, and the performance can even be enhanced by constructing forecasting combinations.

Despite the interesting experimental results, there are some limitations to the current work, which provide future research directions. Firstly, we applied our forecasting models to the Korean film market only. Therefore, our forecasting models should be verified across more countries or markets so as to confirm the utility of the proposed models. Secondly, it would be fruitful to devise screening allocation strategies based on our forecasting results. Because it is not practically possible to control all market participants' decisions, the utility of our forecasting models can be tested by well-designed simulation studies.

## Figures and Tables

**Table 1:** Main contributions of this study.

Categories	Explanatory variables	Forecasting algorithms	Time-horizon
Considering factors	Screening		Three forecasting horizons
	Competition		
	Word-of-mouth (WOM)		

Employed techniques	Genetic algorithm (GA)	Machine learning	
		Forecasting combination	

Interpretation	Two scenarios under three forecasting horizons		
	Scenario 1: competition, WOM, machine learning, and forecasting combination		
	Scenario 2: WOM, competition, machine learning, and forecasting combination		

**Table 2:** Explanatory variable description (*X* denotes the target motion picture whereas *Top*_1_ denotes the motion picture that was ranked first at the box office one day prior to the release day. *Top*_1–5_ denotes the top five movies in terms of box office scores one day prior to release).

Var	Category	Attribute	Description
1	—	Index	Motion picture identifier

2	Screening	*N*_seat^*x*^_*r*_	Number of seats for *X* on the release day

3	Competition	*N*_screen^*y*^_*r*_	Number of screens for *Top*_1_ on the release day
4		*N*_seat^*y*^_*r*_	Number of seats for *Top*_1_ on the release day
5		Sc_share^*y*^_*r*_	Screen share for *Top*_1_ on the release day
6		*N*_screen^*y*^_*r*−1_	Number of screens for *Top*_1_ on one day before release
7		*N*_seat^*y*^_*r*−1_	Number of seats for *Top*_1_ on one day before release
8		Sc_share^*y*^_*r*−1_	Screen share for *Top*_1_ on one day before release
9		IN_number	Number of motion pictures among *Top*_1–5_ that have the same nationality with *X*
10		IN_screen	Number of total screens on the release day for the motion pictures in *Top*_1–5_ that have the same nationality with *X*
11		IN_seat	Number of total seats on the release day for the motion pictures in *Top*_1–5_ that have the same nationality with *X*
12		IN_share	Aggregated screen shares on the release day for the motion pictures in *Top*_1–5_ that have the same nationality with *X*
13		IG_number	Number of motion pictures among *Top*_1–5_ that have the same genre with *X*
14		IG_screen	Number of total screens on the release day for the motion pictures in *Top*_1–5_ that have the same genre with *X*
15		IG_seat	Number of total seats on the release day for the motion pictures in *Top*_1–5_ that have the same genre with *X*
16		IG_share	Aggregated screen shares on the release day for the motion pictures in *Top*_1–5_ that have the same genre with *X*
17		IR_number	Number of motion pictures among *Top*_1–5_ that have the same ratings with *X*
18		IR_screen	Number of total screens on the release day for the motion pictures in *Top*_1–5_ that have the same ratings with *X*
19		IR_seat	Number of total seats on the release day for the motion pictures in *Top*_1–5_ that have the same ratings with *X*
20		IR_share	Aggregated screen shares on the release day for the motion pictures in *Top*_1–5_ that have the same ratings with *X*
21		ID_number	Number of motion pictures among *Top*_1–5_ that have the same distributor with *X*
22		ID_screen	Number of total screens on the release day for the motion pictures in *Top*_1–5_ that have the same distributor with *X*
23		ID_seat	Number of total seats on the release day for the motion pictures in *Top*_1–5_ that have the same distributor with *X*
24		ID_share	Aggregated screen shares on the release day for the motion pictures in *Top*_1–5_ that have the same distributor with *X*
25		Avg_age	The average screening days of *Top*_1–5_ until the release day
26		Rank_screen	Rank of *X* in terms of the number of screens on the release day
27		*N*seat_increase^*y*^	*N*_seat^*y*^_*r*_ − *N*_seat^*y*^_*r*−1_

28	WOM	*N*_SNS_−3_	Total number of SNS mentions posted between three and two weeks prior to the release
29		*N*_SNS_−2_	Total number of SNS mentions posted between two weeks and one week prior to the release
30		*N*_SNS_−1_	Total number of SNS mentions posted during one week prior to the release
31		*N*_emo_−3_	Total number of emotional SNS mentions posted between three and two weeks prior to the release
32		*N*_emo_−2_	Total number of emotional SNS mentions posted between two weeks and one week prior to the release
33		*N*_emo_−1_	Total number of emotional SNS mentions posted during one week prior to the release
34		*N*_pos_−3_	Total number of positive SNS mentions posted between three and two weeks prior to the release
35		*N*_pos_−2_	Total number of positive SNS mentions posted between two weeks and one week prior to the release
36		*N*_pos_−1_	Total number of positive SNS mentions posted during one week prior to the release
37		*N*_neg_−3_	Total number of negative SNS mentions posted between three and two weeks prior to the release
38		*N*_neg_−2_	Total number of negative SNS mentions posted between two weeks and one week prior to the release
39		*N*_neg_−1_	Total number of negative SNS mentions posted during one week prior to the release
40		Tot_SNS	*N*_SNS_−3_ + *N*_SNS_−2_ + *N*_SNS_−1_
41		Avg_SNS_inc	(*N*_SNS_−3_ − *N*_SNS_−1_)/2
42		Weekly_SNS_inc	*N*_SNS_−1_ − *N*_SNS_−2_
43		Tot_emo	*N*_emo_−3_ + *N*_emo_−2_ + *N*_emo_−1_
44		Avg_emo_inc	(*N*_emo_−3_ − *N*_emo_−1_)/2
45		Weekly_emo_inc	*N*_emo_−1_ − *N*_emo_−2_
46		Tot_pos	*N*_pos_−3_ + *N*_pos_−2_ + *N*_pos_−1_
47		Avg_pos_inc	(*N*_pos_−3_ − *N*_pos_−1_)/2
48		Weekly_pos_inc	*N*_pos_−1_ − *N*_pos_−2_
49		Tot_neg	*N*_neg_−3_ + *N*_neg_−2_ + *N*_neg_−1_
50		Avg_neg_inc	(*N*_neg_−3_ − *N*_neg_−1_)/2
51		Weekly_neg_inc	*N*_neg_−1_ − *N*_neg_−2_

**Table 3:** Forecasting model configuration.

Criterion	Identifier	Description
Target	W_1_	Accumulative box office takings in the first week
	W_2_	Accumulative box office takings in the first two weeks
	T	Accumulative box office takings over the entire screening period

Explanatory variables	S	Screening variable only
	SC	Screening + competition variables
	SW	Screening + WOM variables
	SCW	Screening + competition + WOM variables

Forecasting algorithm	MLR	Multiple linear regression
	SVR	Support vector machine
	GPR	Gaussian process regression
	*k*-NN	*k*-nearest neighbor regression
	Comb.	Combining the forecasting results of SVR, GPR, and *k*-NN with equally weighted average

**Table 4:** Tested explanatory variable forecasting combinations for each target.

	MLR	SVR	GPR	*k*-NN	Comb.
S	O				
SC	O				
SW	O				
SCW	O	O	O	O	O

**Table 5:** Selected variables by GA for each forecasting model with MLR as a base learner (all the selected variables are statistically significant at *α* = 0.1).

	Model W_1_	Model W_2_	Model T
Screening + competition (SC)	*N*_seat^*x*^_*r*_ *N*_screen^*y*^_*r*−1_ IN_screen IN_seat IG_screen IG_seat IG_share IR_screen IR_seat IR_share ID_number ID_screen ID_seat	*N*_seat^*x*^_*r*_ Sc_share^*y*^_*r*_ *N*_seat^*y*^_*r*−1_ IN_number IN_seat IG_number IG_screen IG_seat IR_number IR_screen IR_seat ID_number ID_screen ID_seat Avg_age	*N*_seat^*x*^_*r*_ *N*_seat^*y*^_*r*_ *N*_seat^*y*^_*r*−1_ IG_number IG_screen IG_seat IR_number IR_seat ID_number ID_screen ID_seat Avg_age Rank_screen *N*seat_increase^*y*^

Screening + WOM (SW)	*N*_seat^*x*^_*r*_ *N*_emo_−1_ *N*_pos_−1_ Weekly_SNS_inc Tot_emo Weekly_pos_inc	*N*_seat^*x*^_*r*_ *N*_SNS_−1_ *N*_emo_−2_ *N*_neg_−1_ Avg_SNS_inc Tot_pos Tot_neg	*N*_seat^*x*^_*r*_ *N*_emo_−3_ *N*_emo_−2_ *N*_emo_−1_ *N*_pos_−3_ *N*_neg_−1_ Tot_emo Avg_emo_inc Weekly_pos_inc Tot_neg

**Table 6:** Number of selected variables by GA in each category for each forecasting model and algorithm.

Model	Algorithm	Screening	Competition	WOM	Total
Model W_1_	MLR	1	12	12	25
	SVR	1	6	11	18
	GPR	1	16	7	24
	*k*-NN	1	14	10	25

Model W_2_	MLR	1	11	13	25
	SVR	1	9	9	19
	GPR	1	13	10	24
	*k*-NN	1	13	12	26

Model T	MLR	1	11	9	21
	SVR	1	6	12	19
	GPR	1	9	11	21
	*k*-NN	1	9	9	19

**Table 7:** Selected variables by GA for each algorithm and each forecasting model when all screening, competition, and SNS-related variables are considered. The number in each cell indicates whether the corresponding variable is selected for the forecasting model (1: selected, 0: not selected).

Variable	Model W_1_				Model W_2_				Model T			
	MLR	SVR	GPR	*k*-NN	MLR	SVR	GPR	*k*-NN	MLR	SVR	GPR	*k*-NN
*N*_seat^*x*^_*r*_	1	1	1	1	1	1	1	1	1	1	1	1
*N*_screen^*y*^_*r*_	0	0	0	1	0	0	0	0	1	0	1	1
*N*_seat^*y*^_*r*_	0	0	0	0	0	0	0	0	0	1	0	1
Sc_share^*y*^_*r*_	0	0	0	1	0	0	0	1	0	0	0	0
*N*_screen^*y*^_*r*−1_	1	1	1	1	1	1	1	1	0	0	1	1
*N*_seat^*y*^_*r*−1_	0	1	0	1	1	0	0	0	0	1	1	1
Sc_share^*y*^_*r*−1_	1	1	1	0	0	1	0	1	1	0	0	0
IN_number	1	1	0	0	0	0	0	0	1	0	0	1
IN_screen	0	0	1	0	0	1	1	1	0	0	1	0
IN_seat	1	0	1	0	0	1	1	1	0	0	0	0
IN_share	0	0	1	1	0	0	0	0	0	0	0	0
IG_number	0	0	0	0	0	0	1	0	0	0	1	0
IG_screen	1	0	1	1	1	1	1	0	1	1	1	1
IG_seat	0	0	1	1	1	0	0	1	1	0	0	0
IG_share	1	0	1	1	1	0	1	0	0	0	0	0
IR_number	1	0	1	0	1	1	1	1	1	1	0	0
IR_screen	1	0	1	0	0	1	0	1	1	0	0	0
IR_seat	0	0	0	1	1	0	1	0	0	0	0	0
IR_share	1	0	1	1	0	1	1	0	0	1	1	0
ID_number	1	1	1	1	1	0	1	1	1	0	1	0
ID_screen	1	0	1	1	1	0	1	1	1	0	0	1
ID_seat	1	0	1	0	1	0	0	1	0	0	0	1
ID_share	0	0	1	0	0	0	0	1	1	0	0	0
Avg_age	0	1	0	1	1	0	1	0	0	0	0	0
Rank_screen	0	0	0	1	0	1	1	1	0	1	1	1
*N*seat_increase^y^	0	0	1	0	0	0	0	0	1	0	0	0
*N*_SNS_−3_	0	0	0	0	1	0	0	1	0	0	0	1
*N*_SNS_−2_	0	0	0	0	0	1	0	0	0	1	0	0
*N*_SNS_−1_	0	0	0	1	1	0	1	1	0	0	0	0
*N*_emo_−3_	0	1	0	1	1	0	0	1	0	0	0	1
*N*_emo_−2_	1	1	0	0	0	0	1	0	0	1	0	1
*N*_emo_−1_	1	0	1	1	0	1	1	0	1	0	1	1
*N*_pos_−3_	0	0	1	1	0	0	0	1	1	0	1	0
*N*_pos_−2_	1	0	0	0	1	1	1	1	0	1	1	1
*N*_pos_−1_	1	1	0	0	0	1	0	1	1	1	1	0
*N*_neg_−3_	1	1	1	0	0	0	0	1	1	1	0	0
*N*_neg_−2_	0	1	0	1	1	0	0	0	1	1	1	0
*N*_neg_−1_	1	0	0	1	1	1	1	0	0	0	1	0
Tot_SNS	0	0	0	0	1	0	1	1	1	0	0	0
Avg_SNS_inc	0	0	0	0	1	0	0	0	1	0	0	0
Weekly_SNS_inc	1	0	1	1	1	1	1	0	0	0	0	0
Tot_emo	0	0	1	0	0	0	0	1	0	1	1	1
Avg_emo_inc	0	1	0	1	0	0	1	0	0	0	0	1
Weekly_emo_inc	1	1	1	0	1	1	0	0	0	0	1	0
Tot_pos	1	1	0	0	1	1	0	1	1	1	1	1
Avg_pos_inc	0	0	0	0	0	0	0	1	1	1	0	0
Weekly_pos_inc	1	1	1	1	0	0	1	0	0	1	1	1
Tot_neg	1	1	0	0	1	0	1	0	0	0	0	0
Avg_neg_inc	0	0	0	0	0	1	0	1	0	1	0	0
Weekly_neg_inc	1	1	0	1	1	0	0	0	0	1	1	0

*Total*	*25*	*18*	*24*	*25*	*25*	*19*	*24*	*26*	*21*	*19*	*21*	*19*

**Table 8:** Forecasting accuracy in terms of MAPE for each forecasting model and algorithm with different explanatory variables. *∗∗∗*, *∗∗*, and *∗* in the second row in each model denote that the MAPE of the corresponding model is lower than that of (S, MLR) at the significant level of 0.05, 0.1, and 0.2, respectively. Asterisks in the third, fourth, and fifth rows also indicate statistical significance in the aforementioned manner against (SC, MLR), (SW, MLR), and (SCW, MLR), respectively.

Variables	Screening (S)	Screening + competition (SC)	Screening + WOM (SW)	Screening + competition + WOM (SCW)					
Algorithm	MLR	MLR	MLR	MLR	SVR	GPR	*k*-NN	ML average	Combination

*Model W*_1_	0.8383	0.4389	0.7482	0.3515	0.4420	0.3503	0.3175	0.3699	**0.3121**
		(47.64%)^*∗∗∗*^	(10.75%)	(58.07%)^*∗∗*^	(47.28%)^*∗*^	(58.21%)^*∗∗*^	(62.13%)^*∗∗∗*^	(55.87%)	(62.77%)^*∗∗∗*^
				(19.92%)	(−0.70%)	(20.19%)	(27.67%)^*∗*^	(15.72%)	(28.89%)^*∗*^
				(53.02%)^*∗∗*^	(40.93%)^*∗*^	(53.18%)^*∗∗*^	(57.57%)^*∗∗*^	(50.56%)^*∗∗*^	(58.29%)^*∗∗*^
					(−25.75%)	(0.33%)	(9.68%)	(−5.25%)	(11.20%)
									(15.63%)

*Model W*_2_	0.8391	0.6245	0.5325	0.4616	0.3343	0.2975	0.3731	0.3350	**0.3274**
		(25.58%)	(36.54%)	(44.99%)	(60.16%)^*∗*^	(64.54%)^*∗*^	(55.54%)^*∗*^	(60.08%)	(60.98%)^*∗*^
				(26.08%)	(46.46%)^*∗*^	(52.35%)^*∗*^	(40.25%)^*∗*^	(46.36%)	(47.57%)^*∗*^
				(13.30%)	(37.21%)^*∗*^	(44.12%)^*∗∗*^	(29.93%)^*∗*^	(37.09%)	(38.51%)^*∗*^
					(27.58%)	(35.55%)^*∗*^	(19.18%)	(27.44%)	(29.08%)
									(2.26%)

*Model T*	0.5501	0.4702	0.4583	0.3681	0.2666	0.2807	0.3123	0.2865	**0.2599**
		(14.54%)	(16.69%)	(33.10%)	(51.54%)^*∗*^	(48.98%)^*∗*^	(43.23%)^*∗*^	(47.92%)	(52.76%)^*∗*^
				(21.72%)	(43.30%)^*∗*^	(40.30%)^*∗*^	(33.58%)^*∗*^	(39.06%)	(44.72%)^*∗∗*^
				(19.70%)	(41.83%)^*∗*^	(38.75%)^*∗*^	(31.86%)^*∗*^	(37.48%)	(43.29%)^*∗*^
					(27.56%)^*∗*^	(23.73%)^*∗*^	(15.15%)	(22.15%)	(29.39%)^*∗*^
									(9.30%)
